# Organelle-Specific Sensors for Monitoring Ca^2+^ Dynamics in Neurons

**DOI:** 10.3389/fnsyn.2016.00029

**Published:** 2016-09-16

**Authors:** Seok-Kyu Kwon, Yusuke Hirabayashi, Franck Polleux

**Affiliations:** Department of Neuroscience, Mortimer B. Zuckerman Mind Brain Behavior Institute, Kavli Institute for Brain Science, Columbia University Medical CenterNew York, NY, USA

**Keywords:** synapse, mitochondria, endoplasmic reticulum, circuit function, calcium dynamics

## Abstract

Calcium (Ca^2+^) plays innumerable critical functions in neurons ranging from regulation of neurotransmitter release and synaptic plasticity to activity-dependent transcription. Therefore, more than any other cell types, neurons are critically dependent on spatially and temporally controlled Ca^2+^ dynamics. This is achieved through an exquisite level of compartmentalization of Ca^2+^ storage and release from various organelles. The function of these organelles in the regulation of Ca^2+^ dynamics has been studied for decades using electrophysiological and optical methods combined with pharmacological and genetic alterations. Mitochondria and the endoplasmic reticulum (ER) are among the organelles playing the most critical roles in Ca^2+^ dynamics in neurons. At presynaptic boutons, Ca^2+^ triggers neurotransmitter release and synaptic plasticity, and postsynaptically, Ca^2+^ mobilization mediates long-term synaptic plasticity. To explore Ca^2+^ dynamics in live cells and intact animals, various synthetic and genetically encoded fluorescent Ca^2+^ sensors were developed, and recently, many groups actively increased the sensitivity and diversity of genetically encoded Ca^2+^ indicators (GECIs). Following conjugation with various signal peptides, these improved GECIs can be targeted to specific subcellular compartments, allowing monitoring of organelle-specific Ca^2+^ dynamics. Here, we review recent findings unraveling novel roles for mitochondria- and ER-dependent Ca^2+^ dynamics in neurons and at synapses.

## Introduction

Calcium (Ca^2+^) ions govern prevalent physiological processes in various cell types (Rizzuto and Pozzan, 2006; Clapham, 2007). This is especially prominent in excitable cells like neurons where Ca^2+^ influx through the plasma membrane and release of Ca^2+^ from internal stores transduce the effects of changes in membrane polarization and therefore mediate faithful transfer or storage of information over various timescales (milliseconds to minutes/hours). Therefore, regulation of intracellular Ca^2+^ homeostasis is central to the proper function of neuronal circuits. The maintenance of baseline levels of intracellular Ca^2+^ levels is regulated in part through exchangers and pumps such as the plasma membrane Ca^2+^-ATPase (PMCA pump), the Na^+^/Ca^2+^ exchanger (NCX), and the Na^+^/Ca^2+^-K^+^ exchanger (NCKX) which extrude Ca^2+^ through the plasma membrane into the extracellular space. In addition to these mechanisms, intracellular organelles, such as mitochondria and endoplasmic reticulum (ER), are able to regulate cytoplasmic Ca^2+^ ([Ca^2+^]_c_) through mitochondrial calcium uniporter (MCU) and smooth endoplasmic reticulum Ca^2+^-ATPase (SERCA), respectively.

In neurons, mitochondria and ER play important physiological roles via [Ca^2+^]_c_ regulation, thereby diverse synaptic functions including basal synaptic transmission, presynaptic short-term plasticity, and long-term plasticity can be regulated by these organelles (Verkhratsky, 2005; Bardo et al., 2006; Mattson et al., 2008; Vos et al., 2010). In addition, impaired Ca^2+^ homeostasis in the nervous system has been proposed to play an important function in the physio-pathological mechanisms underlying Alzheimer’s disease, Parkinson’s disease, and spinocerebellar ataxia (Verkhratsky, 2005; Mattson et al., 2008; Schon and Przedborski, 2011).

To monitor Ca^2+^ dynamics, various fluorescent Ca^2+^ dyes and genetically encoded Ca^2+^ indicators (GECIs) were developed and applied both *in vitro* and *in vivo*. Also, GECIs tagged with target peptide sequences have allowed imaging of Ca^2+^ dynamics in specific organelles (Rizzuto et al., 1992; Palmer et al., 2004; Palmer and Tsien, 2006).

Previously published reviews have already summarized the usefulness and limitations of various Ca^2+^ sensors and GECIs applied to neuronal and non-neuronal cells (Palmer and Tsien, 2006; Knopfel, 2012; Tian et al., 2012; Rose et al., 2014). In this review, we only describe recently uncovered insights about Ca^2+^ dynamics and its regulation by mitochondria and ER, and we discuss how these organelle-specific Ca^2+^ sensors have been used for the exploration of the role of these subcellular compartments in the regulation of Ca^2+^ homeostasis and synaptic function in neurons.

## Unveiled Synaptic Functions of Mitochondria-Dependent Ca^2+^ Homeostasis

Mitochondrial Ca^2+^ uptake has been studied since the 1950s from studies of rat heart muscle and kidney (Slater and Cleland, 1953; Deluca and Engstrom, 1961). In the nervous system, mitochondria were described at presynaptic terminals and dendrites of various neuronal subtypes using the light and electron microscope (EM) several decades ago (Bartelmez and Hoerr, 1933; Palay, 1956; Gray, 1963; Shepherd and Harris, 1998; Rowland et al., 2000). In axons, mitochondria are short and sparsely distributed, and interestingly, several studies showed that half of presynaptic boutons are occupied by mitochondria (Shepherd and Harris, 1998; Kang et al., 2008). In contrast, dendritic mitochondria have tubular shapes and they are rarely observed in postsynaptic spines in the excitatory neurons (Sheng and Hoogenraad, 2007; Kasthuri et al., 2015).

**Figure 1:**
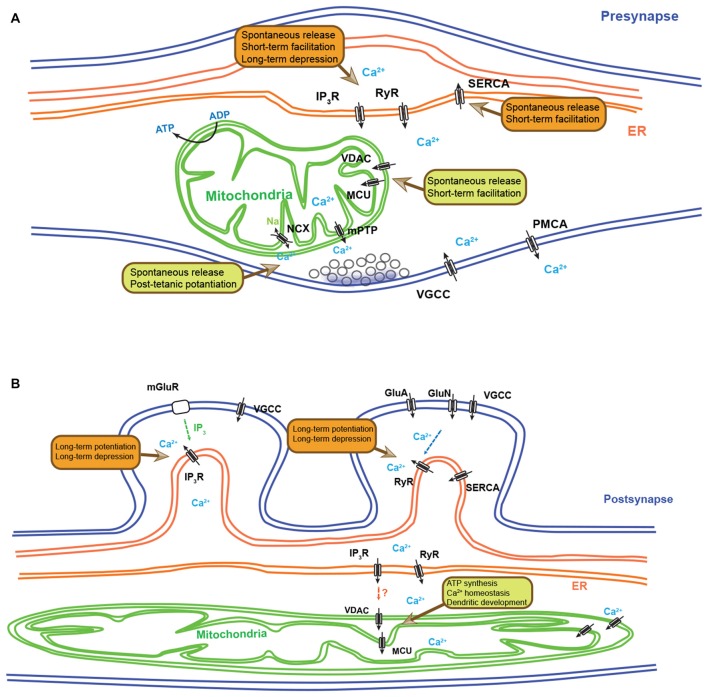
**Synaptic functions regulated by endoplasmic reticulum (ER) and mitochondria-dependent Ca^2+^ homeostasis. (A)** Schematic diagram depicting the presynaptic functions regulated by ER- and mitochondria-dependent Ca^2+^ dynamics. Ca^2+^ release from ER can modulate spontaneous neurotransmitter release, short-term facilitation (STF) and long-term depression (LTD). Ca^2+^ re-uptake by the ER controls the spontaneous release and STF. Presynaptic mitochondria also play important roles in regulating spontaneous neurotransmitter release, STF and post-tetanic potentiation (PTP) through their ability to regulate Ca^2+^ clearance. **(B)** A simplified schematic diagram depicting the postsynaptic functions regulated by ER- and mitochondria-dependent Ca^2+^ dynamics. Ca^2+^ release from ER via IP_3_-induced Ca^2+^ release (IICR) and Ca^2+^-induced Ca^2+^ release (CICR) controls long-term potentiation (LTP) and LTD. In fact, depending on neuronal and synaptic subtypes, IP_3_R and RyR show differential distribution and distinct synaptic functions. Dendritic mitochondrial Ca^2+^ influx can regulate ATP synthesis, Ca^2+^ homeostasis and dendritic development. In non-neuronal cell types, direct Ca^2+^ exchange between ER and mitochondria have been described, but their role in neurons has not yet been documented. IP_3_R, IP_3_ receptor; RyR, ryanodine receptor; SERCA, smooth endoplasmic reticulum Ca^2+^-ATPase; VGCC, voltage-gated Ca^2+^ channel; PMCA, plasma membrane Ca^2+^-ATPase; NCX: the Na^+^/Ca^2+^ exchanger; mPTP, mitochondrial permeability transition pore; MCU, mitochondrial calcium uniporter; VDAC, voltage-dependent anion channel; mGluR, metabotropic glutamate receptor; GluN, NMDA receptor.

At presynaptic boutons and terminals, synaptic vesicle (SV) fusion with the plasma membrane occurs following increase of [Ca^2+^]_c_ following opening of voltage-sensitive Ca^2+^ channels (VSCC) followed by Ca^2+^ binding to sensors like synaptotagmins (Schneggenburger and Neher, 2005; Neher and Sakaba, 2008; Jahn and Fasshauer, 2012; Südhof, 2012). The ability of mitochondria to import Ca^2+^ into the mitochondrial matrix ([Ca^2+^]_m_) plays a role in regulating presynaptic [Ca^2+^]_c_. This has been characterized in various species, neuronal cell types and circuits (Figure 1A). At the *Drosophila* neuromuscular junction (NMJ), the GTPase dMiro mutant lacks presynaptic mitochondria through impaired axonal transport (Guo et al., 2005; Wang and Schwarz, 2009). During prolonged stimulation, these mutants lacking presynaptic mitochondria displayed subtle, but significantly increased presynaptic Ca^2+^ accumulation and display decrease forms of sustained synaptic transmission or synaptic “fatigue” (Guo et al., 2005). *Drosophila* Drp1 mutants also deplete presynaptic mitochondria at NMJ and exhibit elevated presynaptic Ca^2+^ levels in resting and evoked states. However, spontaneous release (mini Excitatory junctional potential, mEJP) was not altered, but the evoked synaptic transmission was impaired during high frequency stimulation, and this defect was partially rescued by ATP (Verstreken et al., 2005) suggesting that mitochondria plays a role in synaptic transmission through their ability to generate ATP through oxidative phosphorylation. Although mitochondrial Ca^2+^ uptake has limited effects on *Drosophila* NMJ neurons, in mammalian NMJ terminals, acute inhibition of mitochondrial Ca^2+^ uptake causes rapid depression of the endplate potential (EPP) and increased asynchronous release (David and Barrett, 2003). Furthermore, in synapses of the mammalian central nervous system (CNS), mitochondria-dependent Ca^2+^ uptake accelerates the recovery from synaptic depression in the calyx of Held (Billups and Forsythe, 2002). Other studies in mammalian hippocampal neurons claimed that impaired mitochondrial anchoring at presynaptic sites increases presynaptic Ca^2+^ during repetitive stimulation and produces short-term facilitation (STF), and insulin-like growth factor-1 receptor (IGF-1R) signaling regulates resting mitochondrial Ca^2+^ level and spontaneous transmission (Kang et al., 2008; Gazit et al., 2016). Although most pharmacological studies employed uncoupling agents as mitochondrial Ca^2+^ influx blocker, which may affect ATP production, these reports support presynaptic control via mitochondrial Ca^2+^ import (Ly and Verstreken, 2006). A recent study demonstrates that presynaptic boutons associated with mitochondria display lower levels of [Ca^2+^]_c_ accumulation than presynaptic boutons not associated with mitochondria (Kwon et al., 2016). Furthermore, acute inhibition of mitochondria calcium import increased [Ca^2+^]_c_ accumulation at presynaptic boutons occupied by mitochondria. In the same study, we demonstrate that this mitochondria-dependent regulation of [Ca^2+^]_c_ plays an important role in regulating presynaptic release properties including spontaneous release, asynchronous release and short-term synaptic plasticity.

In addition to regulation of [Ca^2+^]_c_ clearance, Ca^2+^ release from mitochondria plays important roles at presynaptic sites (Figure 1A). Following the sustained high frequency stimulation, an enhancement of synaptic transmission lasting tens of seconds to minutes is observed and which is called post-tetanic potentiation (PTP; Zucker, 1989). Mitochondrial Ca^2+^ release is suggested as one of the underlying mechanisms for this prolonged enhancement of synaptic transmission. Pharmacological inhibition of mitochondrial Ca^2+^ uptake and release at the crayfish NMJ impaired PTP (Tang and Zucker, 1997; Zhong et al., 2001). Furthermore, similar phenotypes were observed at mouse NMJ and hippocampal mossy fiber synapses with blocking the mitochondrial NCX, which mediates mitochondrial Ca^2+^ release (García-Chacón et al., 2006; Lee et al., 2007).

In contrast to presynaptic boutons and terminals, the postsynaptic function of mitochondrial Ca^2+^ regulation is less well-documented. In mouse hippocampal pyramidal neurons (Li et al., 2004), a minority (<5%) of dendritic spines contains mitochondria. Also, large branched spines in hippocampal CA3 contain mitochondria (Chicurel and Harris, 1992). However, a physiological role of these postsynaptic mitochondria is largely unknown. In general, mitochondria are distributed primarily in dendrite shaft and therefore localized microns away from the postsynaptic density, but might still be able to buffer [Ca^2+^]_c_ mobilized through Ca^2+^-channels and glutamate receptors (Thayer and Miller, 1990; White and Reynolds, 1995; Wang and Thayer, 2002). This mitochondrial calcium import can stimulate tricarboxylic acid (TCA) cycle and might increase ATP production (Kann and Kovács, 2007) and may also regulate other ATP-dependent Ca^2+^ pumps like PMCA and SERCA. While it is still unclear whether or not mitochondria play significant roles in regulating postsynaptic [Ca^2+^]_c_ under physiological conditions of neurotransmission, they might play a role in pathophysiological contexts. For example, neurons lacking LRRK2, a protein associated with Parkinson’s disease, show impaired dendritic Ca^2+^ homeostasis through mitochondrial defects and thought to cause defective mitochondrial depolarization and reduction in dendritic complexity (Figure 1B; Cherra et al., 2013).

Overall, mitochondria-dependent Ca^2+^ clearance and release in neurons plays important physiological and developmental roles pre- and post-synaptically but their functional importance seems to depend on the neuronal subtypes and the structure/size of the pre- and postsynaptic compartments.

## Mitochondrial Ca^2+^-Imaging in Neurons and at Synapses

To investigate organelle-specific Ca^2+^ dynamics, various Ca^2+^ sensors are developed (Table 1). One of the first method developed to monitor mitochondrial Ca^2+^ dynamics was established using rhod-2, a cationic chemical Ca^2+^-binding fluorophore preferentially accumulating in the mitochondrial matrix presumably because of the highly negative membrane potential across the mitochondrial inner membrane (Minta et al., 1989). Then, in the calyx of Held, rhod-2 and rhod-FF (low affinity version) were used to visualize presynaptic mitochondrial Ca^2+^ transient (Billups and Forsythe, 2002). However, these dyes cannot be precisely targeted to these organelles. Therefore, GECIs have recently become the preferred method to image Ca^2+^ in specific organelles including mitochondria. For mitochondrial matrix localization, the targeting presequence of subunit VIII of human cytochrome c oxidase (COXVIII) was tagged to GECIs (Rizzuto et al., 1992). Mitochondria-targeted aequorin (mt-AEQ), a luminescent Ca^2+^ indicator, was first employed to monitor the neuronal mitochondrial Ca^2+^, and this probe showed NMDA-induced mitochondrial Ca^2+^ increase in hippocampal neurons (Baron et al., 2003). However, this probe needs a chemical reaction characterized by a modest turnover rate and has very limited dynamic range (Palmer and Tsien, 2006). Other GECIs have been developed and tested in various neuronal subtypes with the same targeting sequence. Mitochondrial-targeted ratiometric pericam (2mtRP) consists of circularly permutated Enhanced yellow fluorescent protein (cpEFYP) conjugated with Ca^2+^-responsive calmodulin (CaM) and its binding peptide (Nagai et al., 2001; Robert et al., 2001). This probe has a bimodal excitation spectrum and the relative emission intensity is dependent on Ca^2+^-binding. In hippocampal neurons, the use of 2mtRP described mitochondrial Ca^2+^ uptake and also determined cytosolic Ca^2+^ rise upon synaptic activation via dual imaging with cytosolic Ca^2+^ dye (fura-red AM; Young et al., 2008). Other CaM conjugated cpEGFPs called GCaMPs (mito-GCaMP2, 2mtGCaMP6m, and mito-GCaMP5G) were used to monitor axonal mitochondrial Ca^2+^ (Gazit et al., 2016; Kwon et al., 2016; Marland et al., 2016). Both sensors displayed action potential (AP)-dependent mitochondrial Ca^2+^ import. In addition, red fluorescent GECIs by replacing cpEGFP with cpmApple or cpmRuby (mtRCaMP1e and LAR-GECO1.2) revealed mitochondrial Ca^2+^ import simultaneously with cytosolic Ca^2+^ (Akerboom et al., 2013; Wu et al., 2014).

**Table 1:** **Organelle-specific Ca^2+^ sensors in neurobiology**.

Organelle	Sensors	Neuron type	K_d_ for Ca^2+^ (μM)	Excitation used (nm)	Emission filter (nm)	Dynamic Range (F_max_/F_min_, R_max_/R_min_)	Reference
**Mitochondria**	**Dye**	The calyx of Held	0.57, 19	575	590	3.4	Billups and Forsythe (2002)
	Rhod-2, Rhod-FF
	**GECI**
	mito-aequorin	Hippocampal (Hp) neuron	1–2	Luminescence			Baron et al. (2003)
	2mtRP (ratioPericam)	Hp neuron	1.7	Ratiometric, 405/485	535/20	10	Young et al. (2008)
	mito-GCaMP2	Hp neuron	0.195	488	507	5	Marland et al. (2016)
	2mtGCaMP6m	Hp neuron	0.167	488	510	38	Patron et al. (2014), Gazit et al. (2016)
	mtRCaMP1e	Cortical neuron	1.6	572	592.5	6.5	Akerboom et al. (2013)
	LAR-GECO1.2	DRG and Hp neurons	12	561	589		Wu et al. (2014)
**ER**	**Dye**						
	Mag-Fura-2	Sensory neuron	53	Ratiometric, 340/380	510	25	Solovyova et al. (2002)
	**GECI**						
	D1ER	Hp neuron	0.8, 60	FRET, 450	475/40, 535/25	1.6	Zhang et al. (2010)
	erGAP1	DRG neuron, Hp slice	12	Luminescence, 403/470	510	3~4	Rodriguez-Garcia et al. (2014)
	G-CEPIA1er	Cerebellar Purkinje cell	672	488	511	4.7 ± 0.3	Suzuki et al. (2014)
	GCaMPer (10.19)	Cortical neurons	400	490	540/50	14	Henderson et al. (2015)

However, these fluorescent proteins have some limitations, for example, they are affected by pH and mitochondrial matrix pH (pH_m_) can be changed by Ca^2+^ influx (Abad et al., 2004; Poburko et al., 2011; Chouhan et al., 2012; Marland et al., 2016). In addition to this point, [Ca^2+^]_m_ can span broad ranges (0.05–300 μM) depending on cell types and stimulation protocol (Arnaudeau et al., 2001; Palmer and Tsien, 2006). Thus, K_d_ value for Ca^2+^ of mitochondrial GECI should be considered for experimental purposes because high affinity (low K_d_) sensors can be easily saturated by high [Ca^2+^]_m_ and low affinity (high K_d_) sensors may not be sensitive enough to detect small [Ca^2+^]_m_ changes. Several studies reported low affinity mitochondrial Ca^2+^ probes for avoiding saturation (Arnaudeau et al., 2001; Suzuki et al., 2014).

In conclusion, these mitochondria-targeted GECIs allow imaging of mitochondria Ca^2+^ dynamics in neurons and have revealed interesting, synapse-specific properties of mitochondria in the regulation of [Ca^2+^]_c_ and neurotransmitter release properties.

## Regulation of Synaptic Ca^2+^ Dynamics by the Endoplasmic Reticulum

Neurons are among the most polarized cell types in our body and consists of a soma, relatively short dendrites and long axons. ER is found throughout the entire length of neuronal processes, and usually rough ER is prominent in the cell body and proximal dendrites, whereas smooth ER is dominant in distal dendrites, spines and axons (Spacek and Harris, 1997; Verkhratsky, 2005). ER imports and sequesters large amount of Ca^2+^ ([Ca^2+^]_er_ ~500 μM) through SERCA and store-operated Ca^2+^ entry (SOCE) mechanism (Verkhratsky, 2005; Bardo et al., 2006). Ca^2+^ release from ER is mediated by two major mechanisms, called Ca^2+^-induced Ca^2+^ release (CICR) and IP_3_-induced Ca^2+^ release (IICR; Verkhratsky, 2005; Bardo et al., 2006). CICR is caused by the cytosolic Ca^2+^ increase through N-Methyl-D-Aspartate receptors (NMDAR, GluN receptors) and voltage-gated Ca^2+^ channels (VGCCs), whereas IICR is triggered by IP_3_, which is generated via activation of phospholipase C (PLC) depending on metabotropic glutamate receptors (mGluRs) or other receptors like receptor tyrosine kinases (Figure 1).

Ryanodine receptors (RyRs) are involved in CICR, and they have three major subtypes; RyR1, RyR2, and RyR3. All of these isoforms are detected in the brain, and show region-specific expression (Sharp et al., 1993; Furuichi et al., 1994; Giannini et al., 1995; Verkhratsky, 2005; Bardo et al., 2006; Baker et al., 2013). Similar to RyRs, IP_3_ receptor (IP_3_R), which mediate IICR, consist of three isoforms, IP_3_R1, IP_3_R2, and IP_3_R3, but IP_3_R1 is the dominant form in the brain (Sharp et al., 1993, 1999; Verkhratsky, 2005; Bardo et al., 2006; Baker et al., 2013).

Long-term synaptic plasticity is regulated by Ca^2+^-dependent signaling mechanisms such as Ca^2+^/calmodulin-dependent kinase II (CaMKII), calcineurin (a Ca^2+^-dependent phosphatase), protein phosphatase 1 (PP1) and protein kinase C (PKC; Malenka and Nicoll, 1999; Yang et al., 1999; Lüscher and Malenka, 2012). Therefore, Ca^2+^ release from intracellular stores like the ER regulates long-term synaptic plasticity in specific circuits.

In cerebellar Purkinje cell dendrites, mGluR-IP_3_-dependent Ca^2+^ increase is observed during parallel fiber (PF) stimulation and this mediates long-term depression (LTD) of PF-Purkinje cell pathway (Finch and Augustine, 1998; Takechi et al., 1998; Miyata et al., 2000; Wang et al., 2000). At synapses made by hippocampal Schaffer collateral (SC) onto CA1 pyramidal neurons, both long-term potentiation (LTP) and LTD are linked to IP_3_-dependent signaling (Oliet et al., 1997; Nishiyama et al., 2000; Raymond and Redman, 2002; Nagase et al., 2003). In addition, CICR is also observed in CA1 pyramidal neuronal spines, and LTD is abolished in RyR3-deficient mice and following application of RyR inhibitor (ryanodine) although the connection between CICR and LTP is controversial in SC-CA1 pathway (Reyes and Stanton, 1996; Emptage et al., 1999; Futatsugi et al., 1999; Sandler and Barbara, 1999; Kovalchuk et al., 2000; Nishiyama et al., 2000; Raymond and Redman, 2002). Hippocampal mossy fiber pathway (MF, dentate gyrus to CA3) shows IICR- and CICR-dependent LTP and LTD, however, there are conflicting results regarding the underlying mechanisms (Figure 1B; Yeckel et al., 1999; Itoh et al., 2001; Kapur et al., 2001; Mellor and Nicoll, 2001; Lauri et al., 2003; Lei et al., 2003).

Presynaptic ER-dependent Ca^2+^ release is also detected and contributes to changes in neurotransmitter release properties and short-term synaptic plasticity at various inhibitory and excitatory synapses including basket cell to Purkinje cell synapses, hippocampal MF pathway, SC-CA1 and CA3-CA3 pyramidal neuron synapses (Figure 1A; Llano et al., 2000; Emptage et al., 2001; Liang et al., 2002; Galante and Marty, 2003; Lauri et al., 2003; Sharma and Vijayaraghavan, 2003; Unni et al., 2004; Mathew and Hablitz, 2008).

In addition to Ca^2+^ efflux, Ca^2+^ uptake by ER via SERCA pump affects STF at SC-CA1 presynapses and NMJ (Figure 1A; Castonguay and Robitaille, 2001; Scullin and Partridge, 2010; Scullin et al., 2010). Stromal interaction molecules (STIMs) and Orai1, which allow SOCE, are localized to neuronal compartment including dendritic spines, and impaired SOCE alters α-amino-3-hydroxy-5-methyl-4-isoxazolepropionic acid receptor (AMPAR) trafficking, neuronal Ca^2+^ signaling and LTP in CA1 pyramidal neuron and cerebellar Purkinje neurons (Baba et al., 2003; Hartmann et al., 2014; Korkotian et al., 2014; Garcia-Alvarez et al., 2015; Segal and Korkotian, 2015).

## Imaging Neuronal ER Ca^2+^ Dynamics

As mentioned above, ER contains high levels of Ca^2+^, therefore, in order to monitor Ca^2+^ dynamics in the ER lumen, low affinity sensors were employed. Mag-Fura-2, a low affinity membrane-permeable dye (*K*_d_ = 53 μM), has been the first applied for neuronal ER Ca^2+^ measurement, and after loading this dye in the cytoplasm and organelles, cytosolic dye was removed by perfusion with dye-free pipette solution (Solovyova et al., 2002). This allowed for the first time the visualization of caffeine-induced Ca^2+^ release and reuptake in the ER of dorsal root ganglia (DRG) neurons (Table 1).

However, this method can non-specifically label internal compartments, therefore, genetically targeted sensors have been developed more recently for the visualization of ER-derived Ca^2+^ dynamics (Table 1). The signal sequence of calreticulin, a Ca^2+^-binding protein in ER, and an ER retention sequence, KDEL, lead GECIs into the ER lumen, and to optimize for measuring a massive amount of [Ca^2+^]_er_, various mutations were applied to the CaM domain or EF-hand motif of existing GECIs for reducing their Ca^2+^ affinity. A fluorescence resonance energy transfer (FRET)-based Ca^2+^ sensor, D1ER, showed altered ER Ca^2+^ leak function in hippocampal neurons of presenilin double knockout and Alzheimer’s disease model mice (Zhang et al., 2010). Also, bioluminescence-based sensor, GFP-Aequorin protein (GAP), was modified and targeted to ER of DRG and hippocampal neurons, and showed 3- to 4-fold larger ratio change than D1ER (Rodriguez-Garcia et al., 2014). In addition, recently established GCaMP variants for ER Ca^2+^ detection, calcium-measuring organelle-entrapped protein indicator one in the ER (CEPIA1er) and GCaMPer (10.19), characterized ER Ca^2+^ uptake and release in cortical neurons and cerebellar Purkinje cells (Suzuki et al., 2014; Henderson et al., 2015). Interestingly, cerebellar Purkinje cells displayed differential ER Ca^2+^ dynamics in postsynaptic compartment depending on the nature of synaptic inputs (Okubo et al., 2015). These lumenal ER Ca^2+^ indicators also revealed interesting dynamics in dendritic spines, which suggest that [Ca^2+^]_er_ and therefore [Ca^2+^]_c_ can undergo synapse-specific regulation (Suzuki et al., 2014; Henderson et al., 2015).

## Future Perspectives

Recent studies characterized the roles of presynaptic mitochondria and circuit-specific ER Ca^2+^ mobility in dendrites directly via live imaging (Okubo et al., 2015; Kwon et al., 2016), however, organelle-specific Ca^2+^ dynamics at local synapses is only beginning to be explored. Genetically-encoded Ca^2+^ sensors targeted to intracellular organelle and/or to specific synapses as well as functional indicators (like pHluorin-tagged synaptophysin or GluRs) will lead to the identification of synapse- and circuit-specific roles of mitochondria and ER Ca^2+^ in neurons.

MCU has been recently shown to be associated with multiple regulatory proteins, which seems to modify or gate its gating properties and can prevent or enhance mitochondrial Ca^2+^ uptake upon changes in cytosolic Ca^2+^ dynamics (Perocchi et al., 2010; Mallilankaraman et al., 2012; Csordás et al., 2013; Plovanich et al., 2013; Raffaello et al., 2013; Sancak et al., 2013; De Stefani et al., 2015). In addition, MCU activity can be differentially controlled in different tissues (Fieni et al., 2012). Therefore, future investigations should probe the function of this MCU-regulatory complex in neurons and test if MCU and/or MCU-associated proteins can act as neuronal subtype-specific and/or synapse-specific functional modifiers.

In non-neuronal cells, ER and mitochondria establish focal connections which play a key role in Ca^2+^ transfer from ER to mitochondria which has been characterized via intra- and inter-organelle Ca^2+^ imaging (Rizzuto et al., 1993, 2012; Csordás et al., 2010; Kornmann, 2013). This transfer modulates ATP production in mitochondria and may also affect lipid exchange between these two organelles (Voelker, 1990; Cárdenas et al., 2010; Fujimoto and Hayashi, 2011). At present, in neurons, the role of Ca^2+^ translocation between ER and mitochondria is largely unknown. Although immuno-EM images *in vivo* and Ca^2+^ imaging with dyes in respiratory motor neurons suggested ER-mitochondria Ca^2+^ crosstalk, future work will need to establish the context in which ER-mitochondria interface regulates Ca^2+^ dynamics and synaptic function (Takei et al., 1992; Shoshan-Barmatz et al., 2004; Mironov and Symonchuk, 2006).

## Author Contributions

All three authors co-wrote the manuscript.

## Funding

This work was partially funded by support from the NIH-NINDS (R01NS067557, FP), Japan society for the promotion of science fellowship for research abroad and The Uehara Memorial Foundation (YH), and a grant from the Human Frontier Science Program long-term fellowship (S-KK).

## Conflict of Interest Statement

The authors declare that the research was conducted in the absence of any commercial or financial relationships that could be construed as a potential conflict of interest.
